# Monitoring cataract surgical quality

**Published:** 2025-12-02

**Authors:** John Szetu, Lila Raj Puri

**Affiliations:** 1 Medical Director, Fred Hollows Foundation NZ: Regional Eye Centre Honiara, Solomon Islands.; 2 Medical Advisor, Asia: The Fred Hollows Foundation, London, UK.

**Measuring quality helps to identify and correct the causes of poor outcomes.**

**Figure:**
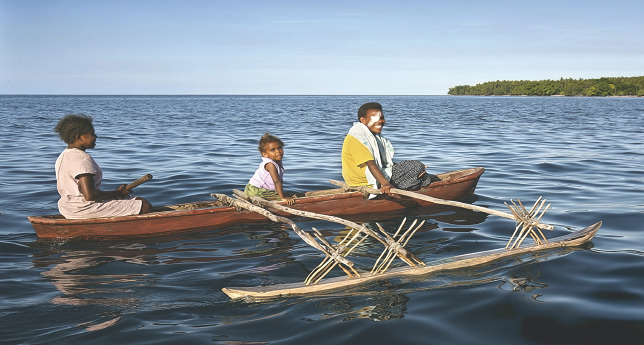
Cataract follow-up is a challenge in the Pacific Islands Region, where many people live in remote areas.

© FRED HOLLOWS FOUNDATION NZ CC BY-NC-SA 4.0

Cataract is the world’s leading cause of sight loss, responsible for 35% of global blindness. The quality of cataract surgery, and resulting patient satisfaction, are the engines that drive a sustainable cataract service. We must be able to measure quality before we can improve it; therefore, it is important to establish a systematic approach for monitoring and evaluating cataract surgical outcomes.

Cataract surgical outcome monitoring (CSOM) is a way to routinely monitor and evaluate cataract surgical outcomes by collecting and analysing preoperative, intraoperative, and postoperative data. These data are analysed, audited, and reported regularly (either monthly, quarterly, or annually) and shared with the hospital team, health and medical authorities, and other interested parties. The team may aim for specific targets to improve surgical quality (see panel).

Information gained during cataract surgical outcome monitoring can help **surgeons** to identify the main causes of poor outcomes and take corrective action to improve. In the last few decades, it has been shown that proactively monitoring the quality of surgical outcomes is associated with improvements in surgical outcomes. **Trainers** can use the data to monitor the ‘surgical learning curve’ of trainees, and **managers** can use the data to inform and monitor continuous quality improvement processes that involve the whole eye care team – e.g. by using the ‘Plan, do, study and act’ (PDSA) cycle, as shown in Figure 1.

**Figure 1:**
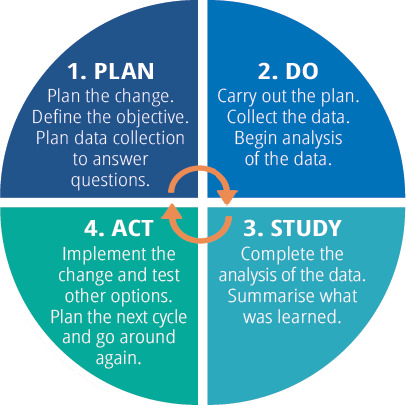
The plan, do, study, and act (PDSA) quality improvement cycle

Implementing a good cataract quality improvement programme, strengthening cataract surgical outcome monitoring, and encouraging eye surgeons to monitor their own results, provides opportunities to create a culture of audit and support, resulting in overall improvement.

It is very important to note that outcome monitoring should not be used to compare individual surgeons or centres. **We should not be tempted to reject patients with complicated cataracts and/or comorbidities for the sake of an audit, as they may benefit greatly from even partial improvement of their sight**.

**Cataract surgical outcome monitoring and WHO recommendations**

Cataract surgical outcome monitoring, and its reporting, varies widely across centres worldwide. Where data are available from lower-income facilities, the outcomes often fall below World Health Organization (WHO) recommendations for cataract surgical outcomes. Meeting these recommendations has become even more difficult now that WHO has raised the cut-off for ‘good vision’ from 6/18 to 6/12. At the time of going to print, the latest WHO recommendation is that ≥ 80% of eyes operated should have postoperative presenting visual acuity (PVA) of ≥ 6/12 (the new recommendations do not contain targets for ‘borderline’ or ‘poor’ vision). **Postoperative presenting visual acuity (PVA)** is defined as postoperative unaided distance visual acuity or, if spectacles or contact lenses are worn to the assessment, with the person wearing these. Postoperative presenting visual acuity should be tested between 4 and 12 weeks postoperatively.

WHO also recommends that countries track postoperative refraction and postoperative complication rates (e.g. posterior capsule rupture with vitreous loss, endophthalmitis, and unplanned return to the operating theatre); these targets should be based on local evidence.

## How to monitor cataract surgical outcomes

Collect the following data for each patient, as routinely as possible:

- Preoperative presenting visual acuity (PVA) at days 1–3, 1 week, and 4+ weeks (see definition of PVA in the panel). Indicate whether the PVA is unaided/uncorrected of if spectacles are worn
- Any improvement in visual acuity (VA) with pinhole
- Any data on pre-existing co-morbidities
- Intraoperative complications, such as posterior capsule rupture
- Postoperative complications, such as corneal oedema or endophthalmitis
- Refractive surprises
- The reasons for poor outcomes: **S**election (pre-existing disease), **S**urgery (surgical complications), **S**pectacles (failure to correct postoperative refractive error), **S**equelae (long-term complications such as posterior capsule opacification, retinal detachment, or corneal complications).

If operating on patients with limited preoperative vision, visual acuity (VA) will be an adequate outcome measure. However, for patients whose vision may be 6/18 or better preoperatively (often those undergoing phacoemulsification), patient-reported outcome measures (PROMs) such as Catquest-9SF or Cat-PROM5 are appropriate validated tools.^4^

Patient experience and patient satisfaction can also be measured to monitor other aspects of the quality of the service (e.g., waiting times in clinic, perioperative pain management, or patient information).

## How to monitor cataract surgical outcomes in low-resource settings

In high-income countries and other well-resourced settings, where local and national outcome monitoring systems are in place, cataract outcomes are often routinely recorded and reported. In lower-resource settings, other methods of outcome monitoring may be needed. For example:

- Data can be collected on paper forms, and/or stored electronically in simple Excel spreadsheets
- BOOST (Better Operative Outcomes Software Tool) can be used. This is an online app which allows surgeons to capture, analyse and monitor cataract outcome data (boostcataract.org)
- Some centres have electronic medical records which may have auditing functions.

## Case study: Cataract surgical outcome monitoring in the Pacific Islands Region

The Pacific Islands Region in Oceania consist of more than a dozen small island states, most of which are low- and/or middle-income countries. In the last two decades, only some of these countries were able to achieve and maintain the previous WHO standard for cataract surgical outcomes: that ≥ 80% of eyes operated should have visual acuity ≥ 6/18.

Because many patients in the region fail to return for follow-up visits, particularly those accessing surgery via outreach programmes, we recommend monitoring visual acuity in the first 1–3 days after surgery. This is based on the recommendations of the PRECOG study,^5^ which demonstrated that visual acuity results 1–3 days after surgery are highly predictive of final vision. When patients are seen at 4+ weeks, we monitor visual acuity against the WHO recommendations (see panel).

Now that WHO have raised the visual acuity cut-off for ‘good vision’ from 6/18 to 6/12, we have chosen to raise our local targets to reflect this change, for both early (1–3 days) and later (4–6 week) postoperative follow-up – see Table 1.

**Table 1:** Locally developed standards for CSOM recommended for the Pacific Islands Region.

**Presenting Visual Acuity (PVA)**	**Early postoperative assessment targets(1–3 days) (Based on PRECOG standards)**	**Late postoperative assessment targets(4+ weeks) (Based on draft WHO recommendations)**
Good (6/6–6/12)	≥ 60%	≥ 80%*
Borderline (<6/12–6/60)	< 35%	n/a
Poor (<6/60)	< 5%	n/a

All other postoperative outcome indicators (postoperative complication rates and postoperative refraction) are based on national evidence-based guidelines. Table 2 contains recommended targets that may be used by countries in the region.

**Table 2:** Other postoperative outcome indicators and recommended targets for the Pacific Islands Region.

**Postoperative outcome indicators**	**Indicator**	**Regional target/benchmark**
Postoperative refraction	Postoperative refraction	± 1 dioptre from target refraction
Postoperative complication rates	Posterior capsule rupture with vitreous loss.	< 5%
	Endophthalmitis incidence	< 0.1%
	Unplanned return to operating theatre	Minimised as per national guidelines

A roadmap for continuous cataract quality improvement is presently in development to assist these small island nations to transition to adopt the new WHO standards. This roadmap includes training, surgical upskilling, and strengthening health systems, and will hopefully serve as a catalyst to enable these countries to transition towards the new WHO recommendations.
